# Effective dose to patients and staff when using a mobile PET/SPECT system

**DOI:** 10.1120/jacmp.v14i3.4250

**Published:** 2013-05-06

**Authors:** Matthew T. Studenski

**Affiliations:** ^1^ Department of Radiation Oncology Jefferson Medical College of Thomas Jefferson University Philadelphia PA USA

**Keywords:** PET, SPECT, effective dose, mobile nuclear medicine

## Abstract

The purpose of this study was to determine the number of weekly acquisitions permissible using a mobile PET/SPECT scanner for myocardial perfusion/viability imaging in an intensive care unit (ICU) based on the effective dose to patients and staff. The effective dose to other patients and staff in an ICU was calculated following recommendations from the American Association of Physicists in Medicine Task Group 108 report (AAPM TG‐108). The number of weekly acquisitions using 555 MBq (15 mCi) Tc‐99m for myocardial perfusion or F‐18 for myocardial viability was determined using the regulatory limits described in the Code of Federal Regulations 10 CFR 20. To increase the number of weekly acquisitions allowed, a reduction in administered dose and portable shielding was considered. A single myocardial perfusion image can be acquired with Tc‐99m each week with a dose reduction to 455 MBq (12.3 mCi) without additional shielding. To acquire a myocardial viability image with F‐18, an activity reduction to 220 MBq (5.9 mCi) is required to meet the regulatory effective dose limit without additional shielding. More than one weekly acquisition can be performed if additional shielding or activity reduction is utilized. A method for calculating dose to patients and staff in an ICU has been developed using conservative assumptions and following AAPM TG‐108. This calculation must be repeated for each individual clinic before any acquisition is performed.

PACS number: 87.57.uq

## I. INTRODUCTION

An economical and mobile bedside PET/SPECT system has been designed that can move within a hospital to image critically‐ill patients such as those in intensive care unit (ICU) or emergency room (ER) settings.^(1)^ Because of their medical condition, these patients cannot easily be transported to a conventional single‐photon emission computed tomography (SPECT) or positron emission tomography (PET) facility. Currently in an ICU or ER, patients with known or suspected severe coronary artery disease typically must be managed without the benefit of myocardial perfusion or viability imaging studies.

The system here was designed to be mobile, economical, and versatile.^(1)^ To overcome the problem of imaging truly immobile patients, the gantry was able to maneuver to a patient's bedside. The system was equipped with dual detector heads to be capable of imaging 140 keV photons (Tc‐99m) in SPECT mode or 511 keV photons (annihilation) in PET mode. NaI $\left(25\times 25\;{\text{cm}}^{2}\right)$ was selected as the scintillating crystal material to reduce costs. This selection resulted in reduced sensitivity at high energies, a limited field‐of‐view, and reduced spatial resolution when compared to a dedicated clinical system. The details of the performance evaluation of the system and a description of the acquisition procedure can be found in the literature.^1^, ^2^ Although conventional systems can achieve better performance parameters, these conventional systems are often not a viable option for patients in an ICU or emergency room or are not economically feasible.

The clinical feasibility study of this mobile system did not address the radiation dose to patients and staff in the surrounding areas during an acquisition. A typical nuclear medicine suite is designed with shielding, and access to the room is controlled to prevent people from entering and receiving unnecessary dose. In the environment this mobile system is used, the rooms are not shielded and access is not restricted. To account for this, the dose to patients and staff involved in an acquisition in an ICU was calculated using recommendations from the American Association of Physicists in Medicine (AAPM) Task Group 108 (TG‐108).^(3)^ Activity reduction and shielding^(4)^ were considered as options to reduce the dose to patients and staff and to allow for multiple acquisitions weekly.

## II. MATERIALS AND METHODS

### A. Mobile system

**Figure 1:**
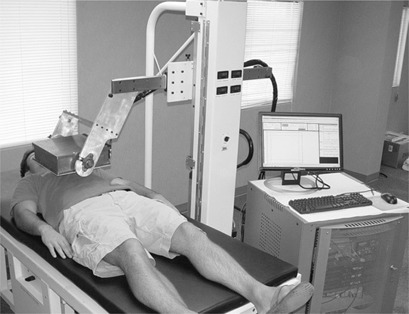
Mobile PET/SPECT system shown in a bedside position. Note the electronics rack to the right and a single detector positioned for SPECT imaging.

The mobile system can be seen in Fig. 1. The system can be positioned at the bedside of the patient and an acquisition can be performed in SPECT or PET mode. SPECT mode uses one or two detector heads with a parallel hole or pinhole collimator. Different collimators have been designed for cardiac perfusion imaging using Tc‐99m at 140 keV and myocardial viability imaging using F‐18 fluorodeoxyglucose (FDG) at 511 keV. The system can also operate in PET mode without physical collimation with the detectors in an opposed orientation.

### B. Regulatory limits

Regulatory limits for radiation doses are established in the code of federal regulations 10 CFR part 20. The effective dose equivalent in an unrestricted area must be less than 1 milli‐Sievert/year (mSv/year) or 20 micro‐Sieverts (μSv) in any one hour, implying a weekly limit of 20 μSv to be conservative.^(5)^ As the typical ICU stay is only about four days,^(6)^ as long as the dose does not exceed 20 μSv in any one hour, the limit to adjacent patients could be interpreted to be 1 mSv, although in this calculation conservative assumptions are used. The occupational dose limit is higher at 50 mSv/year (a weekly limit of 1 mSv).^(7)^ This is the limit in a restricted area such as a designated nuclear medicine suite. Acquisitions with this system will be in an ICU environment with patients and staff who are members of the general public, so the regulatory limits for an unrestricted area must be considered.

### C. Effective dose calculation

Although there are similar aspects between a nuclear medicine procedure in a typical nuclear medicine suite and in an ICU, there are differences that need to be accounted for in the dose calculation. In a typical nuclear medicine procedure, the patient receives an injection containing the radiopharmaceutical and then is held in a room for about an hour while the organ of interest metabolizes the compound. The patient is then brought to the scanner and the acquisition is performed. During this time, other patients and the staff have minimal interactions with the patient. Following the acquisition, the patient is released with no further exposure to other patients and staff. With the mobile system, the patient is not moved throughout the entire procedure and staff might have to interact with the patient to monitor vital signs or administer medications. Furthermore, the patient is not released after the acquisition and will remain in bed.

A framework for calculating exposure and shielding requirements for a traditional nuclear medicine suite has been developed in AAPM TG‐108.^(3)^ As this mobile system is not used in a typical suite and patients are managed differently, the recommendations in TG‐108 have been modified accordingly.

The effective dose is calculated in three parts: 1) one‐hour uptake period, 2) one‐hour acquisition period, and 3) a decay period. For the initial dose calculations, the calculation assumes there is no shielding and the patient is administered a typical dose of 555 mega‐Becquerels (MBq) (15 mCi).

**Figure 2:**
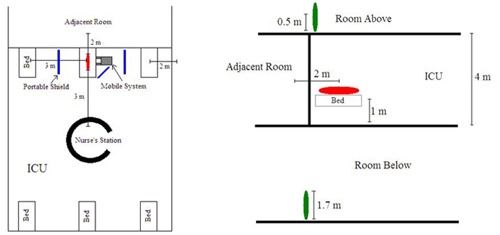
Top and side views of the distances assumed in the ICU for the dose calculation.

Figure 2 shows the distances assumed in a typical ICU.^(3)^ The calculation accounts for three groups of people who are exposed: 1) other immobile patients in the ICU and surrounding rooms, 2) public‐limit staff working in the ICU, and 3) occupational‐limit staff working in the ICU and performing the acquisition.

#### C.1 Dose rate constants

The effective dose rate constant, Γ, from the 140 keV Tc‐99m photons is much lower than for 511 keV F‐18 annihilation photons ($F-18:0.143\;\mu \text{Sv}\;m^{2}/\text{MBq}\;h$ ^(3)^ and $\text{Tc}-99m:0.021\;\mu \text{Sv}\;m^{2}/\text{MBq}\;h$ ^(8)^). For the dose calculation with F‐18 in TG‐108, the dose rate is calculated from the effective dose rate constant and is reduced to account for attenuation in the body. Approximating the patient as a point source is valid for a shielding calculation, but not in an ICU environment case where distances are 1 m or less and activity could be concentrated in superficial organs. Consequently, to be conservative, there is no reduction in the effective dose rate constant for either F‐18 or Tc‐99m in this calculation.

#### C.2 Uptake period

The effective dose (D) to a point d meters from a patient during the initial uptake period of 1 hour is defined in Eq. (1) using the method described in TG‐108 modified with the appropriate dose rate constant, Γ, mentioned in section C.1 above.^(3)^

(1) $$D(\mu \text{Sv})=\Gamma \frac{\mu \text{Sv}\;m^{2}}{\text{MBq}\;h}\times A_{0}(\text{MBq})\times R\times \frac{\text{uptake}\;\text{time}\;(h)}{d{\left(m\right)}^{2}}$$

The effective dose depends on the initial activity $A_{0}$, the uptake time (1 hour), the distance from the patient, d, and a dose reduction factor, R, to account for radioactive decay averaged over each time period.^(3)^ The dose reduction factor is calculated using Eq. (2) as in TG‐108.^(3)^

(2) $$R=1.433\times \left(\frac{\text{Radioisotope half life}\;(h)}{\text{Lengh of time period}\;(h)}\right)\times \left[1‐\text{exp}\left(\frac{‐0.693\cdot \text{Length of the time period}\;(h)}{\text{Radioisotope half life}\;(h)}\right)\right]$$

#### C.3 Acquisition period

In TG‐108, for the acquisition period calculation, a dose reduction of 15% is assumed due to the excretion of some of the radiopharmaceutical through urine during the uptake period (excretion is usually forced). This is not possible with patients in an ICU, so this 15% decrease cannot be accounted for, although the decay that occurred up to this point, F, can be calculated using a decay factor (Eq. (3)). The dose D, to a point d meters from the patient during the acquisition time is calculated using Eq. (4). R is again used to account for the decay during each calculation period.

(3) $$F=\text{exp}\left(‐0.693\times \frac{\text{time since injection}\;(h)}{\text{Radioisotope halp life}\;(h)}\right)$$

(4) $$D(\mu \text{Sv})=\Gamma \frac{\mu \text{Sv}\;m^{2}}{\text{MBq}\;h}\times A_{0}(\text{MBq})\times R\times F\times \frac{\text{acquisition time}\;(h)}{d{\left(m\right)}^{2}}$$

The acquisition time with this system is around 1 hour, but could be extended into the decay period, if required.

#### C.4 Decay period

Unlike a normal nuclear medicine scan, a patient in the ICU is not released following the acquisition. During the decay period, the accumulated dose is calculated every hour after the acquisition period until the dose rate at 0.5 m is less than 1 μSv/h (about 15 hours for F‐18 and 29 hours for Tc‐99m). This level is chosen as it is below the dose rate of a designated radiation area. Including the remaining integrated dose after this time does not significantly affect the result, either (remaining integrated dose ∼1% of total effective dose). Excretion is not accounted for, so this time could be reduced for a less conservative calculation. The effective dose during the decay time is calculated using Eq. (4) where F is modified to account for the reduction in dose due to decay up to the start of the calculation time and R still accounts for the reduction during the calculation time (one‐hour time period).

### D. Dose to patients and staff

As patients and staff have different involvement in the acquisition in terms of distance and time near the patient, the total effective dose accumulated every hour is calculated at different distances from the patient through the uptake, acquisition, and decay periods. This allows the total effective dose to be integrated over the entire procedure for each specific group as a function of distance from the patient and time spent near the patient.

#### D.1 Other patients

To be conservative, it is assumed that all other patients in the ICU and adjacent rooms will not be moved from their beds during the entire acquisition from uptake through decay. The effective dose is the integrated dose over all three periods during the acquisition. The total effective dose is compared to the 20 μSv weekly limit to determine the number of cases that can be performed per week.

#### D.2 Public‐limit ICU staff

For those staff in the ICU who are not trained occupational workers, it can be argued that they should not approach the injected patient at all, but it is also realized that they must still perform their duties in the ICU near the injected patient. For the effective dose calculation for public‐limit staff in the ICU, ten‐hour shifts are assumed. A ten‐hour shift accounts for a staff member working overtime or a long ICU shift. The effective dose is only integrated over the first ten hours of the acquisition accounting for the worst case scenario where a shift starts during the uptake period when the dose rate is at its maximum. The total effective dose is compared to the weekly limit of 20 μSv to determine the number of cases that can be done each week. This is again a conservative approach, as the staff member will most likely not be in the ICU for the entire shift.

#### D.3 Occupational‐limit staff

It is assumed that any staff directly involved in the patient's care is an occupationally trained worker. This includes the ICU staff and the staff operating the mobile system. As these individuals must approach the patient to monitor vital signs and operate the mobile system, they will be at a distance of 0.5 m from the patient for a certain amount of time each hour. It is assumed that for the remainder of that time, they are at a distance of 4 m from the patient. With these occupational‐limit staff, it is necessary to determine the amount of time each hour they can monitor the patient and still meet the regulatory weekly dose limit of 1 mSv. As with the public‐limit staff, a ten‐hour shift is assumed.

### E. Dose reduction options

There are three ways to reduce dose: reducing exposure time, increasing distance, and increasing shielding. For other immobile patients in an ICU, it may not be possible to reduce the exposure time or increase the distance, but portable shielding can be utilized or the initial activity administered to the patient can be reduced. The effective dose to patients and public‐limit staff at a distance of 2 m is calculated assuming a reduction in initial administered activity.

Without extensive construction, it is difficult to shield adjacent rooms, but it is possible to utilize portable shields. As a baseline to study the effect of shield thickness, the assumption is that immobile patients and public‐limit staff in the ICU are 2 m from the injected patient. Two different portable radiation shields are considered: one for other patients, and one for staff in the ICU. There are commercially available portable shields that can be wheeled into place between patient beds or next to the bed to shield the staff directly treating the patient. A shield could also be constructed from lead and attaching wheels. For this calculation, the shield design is a lead sheet on wheels. The shield for other patients is 1.8 m wide by 1.8 m tall (6 ft×6 ft). This shield could be rolled into place between patients beds both for dose reduction and for privacy. The lead shield for the workers is 0.9 m wide and 1.8 m tall (3 ft×6 ft). This shield is on wheels to allow for easy access to the patient for detector positioning and monitoring of the patient's vital signs. The effective dose is calculated using shield thicknesses of 1 mm up to 15 mm for both F‐18 and Tc‐99m. The reduction in dose is compared to the potential weight of each shield design

## III. RESULTS

### A. Dose to other patients

**Figure 3:**
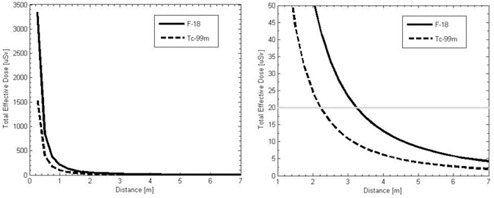
Total effective dose per acquisition integrated over all time periods as a function of distance from the patient with both F‐18 and Tc‐99m. Full range of doses (on the left) and zoomed in to show the dose with respect to the 10 CFR 20 weekly limit (on the right).

The total effective dose at various distances for both F‐18 and Tc‐99m can be seen in Fig. 3. With the regulatory limit of 20 μSv per week, it is possible to perform one exam per week with an immobile patient at least 2.25 m away with Tc‐99m and with a patient at least 3.25 m away with F‐18 without exceeding the regulatory dose limit.

The first two hours following the injection are the most critical to ensure that no personnel remain close to the patient for an extended period of time. Comparing these results to Fig. 2, it is possible for a patient to be at a distance of 2 m in an adjacent room. This means that with F‐18 and possible Tc‐99m, a patient in the adjacent room would exceed the regulatory limit, so some means of reducing the effective dose rate must be implemented.

### B. Dose to public‐limit staff

**Figure 4:**
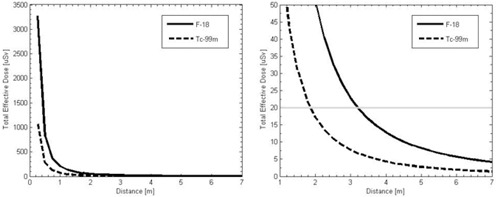
Total effective dose per acquisition integrated over the first ten hours of the procedure as a function of distance from the patient with both F‐18 and Tc‐99m. Full range of doses (on the left) and zoomed in to show the dose with respect to the 10 CFR 20 weekly limit (on the right).

For the public‐limit staff in the ICU not directly involved with the patient, the total effective dose as a function of distance from the patient over the first ten hours of the procedure can be seen in Fig. 4. To meet the regulatory limit, the staff member must be at least 2 m from the patient for a procedure with Tc‐99m and 3.25 m from the patient for a procedure with F‐18. Comparing these results to Fig. 2, public‐limit staff in rooms above and below the ICU would be at a distance of about 4 m, thereby meeting the regulatory limit. Public‐limit staff working the nurse's station in the ICU would be at a distance of about 3.5 m, which would also meet the regulatory limit for procedures with either isotope. For these public‐limit staff members, one acquisition could be done per week in the ICU without exceeding the regulatory dose limit.

As with the immobile patient dose calculation, it would be possible for a public‐limit staff member to be in an adjacent room at a distance of 2 m from the patient. A single acquisition per week would not exceed the regulatory limit with Tc‐99m, but the staff member would exceed the dose limit for a procedure with F‐18; therefore, this effective dose must be reduced.

### C. Dose to occupational‐limit staff

**Figure 5:**
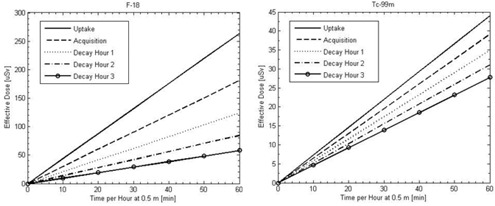
Effective dose for occupational‐limit staff as a function of the time spent at 0.5 m from the patient for acquisitions with both F‐18 and Tc‐99m.

**Figure 6:**
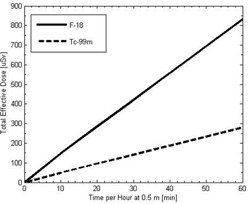
Total effective dose during a ten‐hour shift for occupational‐limit staff assuming that the time spent at 0.5 m is the same every hour and the rest of the time is spent at least 4 m from the patient with both F‐18 and Tc‐99m.

Figure 5 shows the effective dose as a function of time per hour spent at a distance of 0.5 m from the patient. Figure 6 shows the total effective dose assuming the same amount of time is spent near the patient each hour, plus the effective dose accumulated at a distance of 4 m over the same ten‐hour period (12.8 μSv with F‐18 and 4.3 μSv with Tc‐99m). With a weekly regulatory limit of 1 mSv, the occupational‐limit worker would be limited to a single F‐18 acquisition or three Tc‐99m acquisitions per week if the entire time was spent at 0.5 m. If only 30 minutes each hour was spent at 0.5 m, then two F‐18 or seven Tc‐99m acquisitions could be done each week.

### D. Dose reduction techniques

It is possible to perform one Tc‐99m procedure per week and not exceed the regulatory limit with the previous assumptions, but this is not the case for an acquisition with F‐18. Depending on the layout of the hospital, the dose to immobile patients or staff in an adjacent room could exceed the regulatory limit. The limits would also be exceeded if multiple procedures were done each week, so either the activity administered to the patient needs to be reduced or portable shielding must be used.

#### D.1 Activity reduction

**Figure 7:**
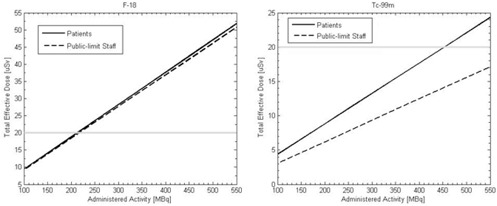
Total effective dose to other immobile patients and public‐limit staff at 2 m from the patient as a function of injected activity.

Figure 7 demonstrates the effect of reducing the activity administered on the total effective dose to immobile patients or public‐limit staff members that are in an adjacent room at a distance of 2 m. The dose to an immobile patient is integrated over the entire procedure and the dose to public‐limit staff is integrated over the first ten hours of the procedure. To reduce the dose below the regulatory limit for these individuals, the administered activity for acquisitions with F‐18 must be reduced to about 220 MBq (5.9 mCi) and 455 MBq (12.3 mCi) for Tc‐99m. A reduction in activity to about 225 MBq (6.1 mCi) would allow two acquisitions to be done each week with Tc‐99m.

#### D.2 Portable shielding

**Figure 8:**
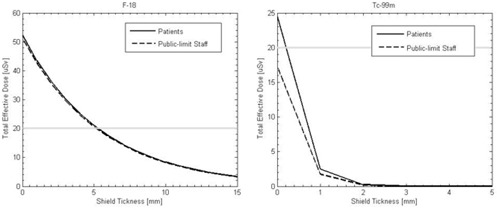
Total effective dose to other immobile patients and public‐limit staff as a function of shield thickness for both F‐18 and Tc‐99m procedures.

Figure 8 shows the total effective dose for a typical activity of 555 MBq to public‐limit ICU staff and other patients at a distance of 2 m with different thicknesses of lead shielding. Table 1 shows the weights of both shields for the different lead thicknesses. Due to the higher attenuation coefficient of lead at lower energies, the shield is more effective for Tc‐99m. Use of the shield for patients is not always necessary for keeping the dose below the regulatory limit, but it is important to follow the ALARA principle (as low as reasonably achievable). To reduce the dose below the regulatory limit for public‐limit staff and patients, with an F‐18 procedure, a shield thickness of 5 mm is required. A 1 mm thick shield would be sufficient for Tc‐99m.

**Table 1:** Approximate portable shield weight for varying thicknesses of lead.

	*Weight [kg] (lbs)*	
*Thickness [mm]*	*Patient Shield* (6 ft×6 ft)	*Staff Shield* (3 ft×6 ft)
0	–	–
1	33 (72)	16 (36)
3	122 (270)	61 (135)
6	245 (540)	122 (270)
13	540 (1080)	245 (540)

## IV. DISCUSSION

Although reducing the activity administered to the patient is a simple means of reducing dose, there will be degradation in the quality of the image. The main cause of image degradation would be increased noise from reduced counts in the projection images.^(2)^ One way to alleviate this effect is to increase the scan time. Since the patient is immobile and a long decay time was assumed, a longer scan time with lower injected activity would reduce the effective dose to surrounding areas while providing an increase in image quality. For FDG specifically, the fact that these patients are immobile means that there is reduced uptake in skeletal muscle and greater uptake in the myocardium, increasing the signal to noise ratio in the image. Because of the complexity of the uptake of the radiotracers, the total effect of reducing the activity on the overall image quality cannot be determined until further studies are done in a clinical environment.

This procedure, based on a conservative set of assumptions for patients and staff, could be an overestimate for some hospital layouts. In some cases, the limiting dose might be to the public‐limit staff in the ICU. The dose to the occupational‐limit staff should not be a limiting factor, but if this time near the patient is reduced, additional acquisitions could be performed. With portable shielding or activity reduction, the potential number of weekly acquisition increases.

It is important to note that the dose close to the patient is above the regulatory limit, especially during the uptake period, and precautions should be made such that that no individual is close to the patient for an extended period of time. From 10 CFR 20, an area is a high‐radiation area and access must be controlled if the dose rate is greater than 1 mSv/h at 30 cm from the source.^(9)^ For a 555 MBq injection, the dose rate at 30 cm is 0.8 mSv/h for F‐18^(3)^ and 0.13 mSv/h for Tc‐99m.^(8)^ This means access does not need to be restricted, but signs do need to be posted declaring the area a radiation area (an area in which the dose rate at 30 cm from the source is at least 0.05 mSv/h^(10)^). To make sure staff maintain proper distances, lines can be drawn on the floor. Removal and proper disposal of the patient's urine is necessary and will help reduce the dose in the surrounding areas, although this is not accounted for in this calculation. For any procedure to be performed in an ICU, radiation training is recommended for the public‐limit staff.

This calculation makes several assumptions to prove the concept of acquiring a tomographic image in an ICU environment. Before any clinical procedures are performed, this calculation must be repeated to account for the specific layout of the hospital and ICU and for the distances personnel will be from the injected patient. The method used here does provide a template for future dose calculations with this mobile system in an ICU environment.

## V. CONCLUSIONS

A procedure for calculating the effective dose to patients and personnel in an ICU using a mobile PET/SPECT scanner has been developed following recommendations from AAPM TG‐108. Using conservative assumptions, it is possible to acquire a single myocardial perfusion image with Tc‐99m each week using an activity of 455 MBq (12.3 mCi) without any extra shielding. To acquire a myocardial viability image with F‐18, an activity reduction to 220 MBq (5.9 mCi) is required to meet the regulatory effective dose limit without shielding. This calculation must be repeated for each individual clinic before any acquisition is performed.

## ACKNOWLEDGMENTS

The author would like to thank Dr. David Gilland and Dr. David Hintenlang of the University of Florida for their insightful comments into the development of this manuscript.
